# Effective Rabi frequency in ultrafast semiconductor lasers: self-starting harmonic frequency combs

**DOI:** 10.1038/s41377-026-02342-1

**Published:** 2026-07-10

**Authors:** Carlo Silvestri, Franco Prati, Massimo Brambilla, Mariangela Gioannini, Lorenzo Luigi Columbo

**Affiliations:** 1 Institute of Photonics and Optical Science (IPOS), School of Physics, The University of Sydney, Sydney, NSW 2006 Australia; 2 Dipartimento di Scienza e Alta Tecnologia, Università dell’Insubria, Via Valleggio 11, 22100 Como, Italy; 3 Como Lake Institute of Photonics, Università dell’Insubria, via Valleggio 11, 22100 Como, Italy; 4 Dipartimento Interateneo di Fisica, Politecnico di Bari and CNR-IFN, UOS Bari, Italy; 5 Dipartimento di Elettronica e Telecomunicazioni, Politecnico di Torino, 10129 Torino, Italy

**Keywords:** Quantum cascade lasers, Semiconductor lasers

## Abstract

Optical frequency combs have become a key research topic in optics and photonics. A peculiar comb state is the Harmonic Frequency Comb (HFC), where optical lines are spaced by integer multiples of the cavity’s free spectral range. The spontaneous formation of HFCs has recently been observed in semiconductor lasers with fast gain recovery, such as Quantum Cascade Lasers (QCLs), although the underlying physical mechanism remains unclear. In this work, we provide a physical interpretation for the formation of HFCs in QCLs, based on a resonance phenomenon between an effective Rabi frequency and a mode of the laser cavity. This is corroborated by the results of the numerical integration of the Effective Semiconductor Maxwell-Bloch Equations (ESMBEs) used to describe the multimode laser dynamics, as well as by the linear stability analysis of the continuous-wave emission.

## Abstract (web summary)

We derive an Effective Rabi Frequency (ERF) for semiconductor lasers and explain harmonic frequency comb formation in quantum cascade lasers as a resonance between ERF and a cavity mode, that makes the latter undamped.

## Graphical abstract

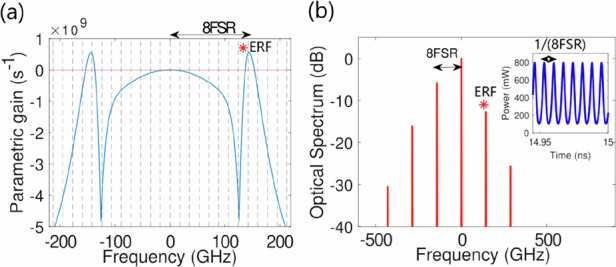

## Introduction

Optical Frequency Combs (OFCs) are spectra associated with regular dynamical regimes exhibiting equally spaced optical lines, which adhere to a fixed phase relation^1^. In the time domain, they correspond to a periodic signal generally in the form of optical pulses. OFCs serve as a precise frequency ruler, making them pivotal for high-precision measurements. In fact, their introduction has driven significant advancements in the fields of metrology, spectroscopy, and optical communications^2–6^.

The last decades have witnessed a revolution in the study and development of comb sources, marked by the emergence of two typologies of devices with significant impact on chip-scale integration: optically pumped Kerr microresonators^7^ and fast semiconductor lasers, such as Quantum Cascade Lasers (QCLs)^8^ and Quantum Dot Lasers (QDLs)^9^. These systems share the same underlying mechanism for comb generation, based on an efficient cascading four-wave mixing process^10–13^.

QCLs are unipolar semiconductor lasers emitting in the mid-infrared (mid-IR) and terahertz (THz) region of the electromagnetic spectrum, characterized by a picosecond carrier lifetime dominated by fast phonon scattering that makes QCLs ultrafast devices^14^. This property, together with a small but finite value of the Linewidth Enhancement Factor *α* (LEF) which characterizes semiconductor active media, allowed for the demonstration of frequency combs in QCLs in the mid-IR and in the THz regions^10,11,15^. This evidence has not only paved the way for transferring the aforementioned application advances to these spectral regions^11,16,17^, but has also held significant importance from a fundamental perspective. In fact, QCLs exhibit a variety of comb states whose distinctive properties have been progressively clarified through theoretical advancements in recent years. In particular, QCL combs in the Fabry-Perot (FP) configuration display a coexistence of shallow Amplitude Modulation (AM) and Frequency Modulation (FM) behavior^18–23^. On the other hand, in the unidirectional ring configuration, OFCs are associated with AM localized and global structures in the form of solitons, and Turing rolls^24–29^ in analogy with the case of Kerr microresonators^12,30^. Close to the lasing threshold, the formation of OFCs in ring QCLs is accurately described by the Complex Ginzburg-Landau Equation (CGLE)^25,29^ and, in driven systems, by the Generalized Lugiato-Lefever equation^28^. We observe that the QCL ring configuration is of particular interest for integrated photonic applications^29^.

Another peculiar feature of QCLs is their ability to emit self-starting Harmonic Frequency Combs (HFCs), characterized by an optical line spacing that is an integer multiple > 1 of the cavity Free Spectral Range (FSR). The first observation of harmonic states in QCLs dates back to 2016 in FP mid-IR devices^31^, and the coherent nature of these regimes was demonstrated the following year^32^. Subsequently, HFCs have also been reported in the THz range^33,34^ and in the ring configuration^35^, emerging as characteristic states of QCLs with compelling applications in microwave generation, pump-probe spectroscopy, and broadband spectroscopy^36^. This has motivated the development of various configurations to induce and control HFCs, such as radiofrequency^33^ and optical injection^37^, optical feedback^38,39^, laser temperature tuning^36^, surface and intracavity defects^40,41^.

Although many aspects of OFCs formation in QCLs have been understood from a theoretical perspective, the physical origin of self-starting HFCs remains not completely understood^17,42^. Early theoretical studies on the multi-mode instability underlying frequency comb formation in an FP laser were based on Maxwell-Bloch equations for two-level lasers. Spatial Hole Burning (SHB), linked to the presence of a standing wave pattern and the associated carrier grating in the laser cavity, was considered instrumental for generating multimode emission close to the lasing threshold^43–45^. It was shown that in an FP laser, the single-mode state can become unstable in two ways: by a phase (FM) instability or by an amplitude (AM) instability, and in this framework, HFCs in QCLs were associated with the existence of peaks in the nonlinear parametric gain displaced by multiples of the cavity FSR^31,46^. This mechanism bears some analogies with the well-known Risken-Nummedal-Graham-Haken (RNGH) instability affecting two-level lasers and consisting in the amplification of the system Rabi frequency when it is resonant with a cavity mode^47,48^. With our notations, the Rabi frequency is proportional to $\sqrt{X}$, where *X* is the dimensionless field intensity in the stationary state. All these studies were based on the assumption of laser operating close to the threshold, which allows to keep only the first harmonics in the population grating. In^49,50^ that assumption was removed, and this allowed to clarify that the RNGH instability in a FP laser occurs only for extremely high and unrealistic values of the pump and, more importantly, that the instability close to threshold has a different origin. Indeed, the boundary of the instability domain observed close to threshold scales as *X*^1/4^ instead of $\sqrt{X}$^43,51^.

In this letter, we study the phenomenon of HFC formation using a set of Effective Semiconductor Maxwell Bloch Equations (ESMBEs)^24,52^ for a unidirectional ring QCL where both the periodic boundary conditions and the absence of SHB simplify system dynamics and allow for an analytical approach.

ESMBEs realistically account for the radiation-matter interaction peculiarities in a semiconductor laser, characterized by a non-zero *α* factor which provides the well-known asymmetries in the medium gain and dispersion. We remark that this approach played a fundamental role for the prediction and explanation of OFCs formation for *α* > 1 and close to threshold in unidirectional ring QCLs^24^ where they are associated with the presence of the homoclons typical of the Benjamin-Feir (BF) instability^53^. In this work, we confirm and extend these results, but we also show that for *α* < 1, there is an RNGH-type instability further from the threshold associated with an undamped effective Rabi frequency.

On this basis, we provide a theoretical explanation for the origin of self-starting HFCs. In particular, we show that HFCs spontaneously emerge from a resonance between a cavity mode and an intrinsic oscillation frequency of the material variables (carrier density and macroscopic medium polarization). This frequency shows a dependence on the square root of the intensity, a renowned fingerprint of the Rabi frequency in several coherent matter-field phenomena, so we named it Effective Rabi Frequency (ERF). Remarkably, we could show that the resonance triggers a Continuous-Wave (CW) instability, leading to the spontaneous onset of an HFC whose order is equal to the number of cavity modes (or FSR) between the ERF and the unstable CW frequency. Our theoretical explanation is corroborated by a Linear Stability Analysis (LSA) of the full ESMBEs model that shows good agreement between the ERF and the frequency of maximum parametric gain. Our numerical simulations confirm and substantiate the predictions of the previous analysis. Similar results on HFCs formation were predicted recently in unidirectional ring QDLs at telecom wavelengths using a two-level system approach and inhomogeneous broadening^54^.

## Results

We start from the ESMBEs that describe the dynamics of a multimode unidirectional ring QCL^24^:

1 $$\frac{\partial F}{\partial \eta }+\frac{\partial F}{\partial t}=\sigma [-F-P]$$

2 $$\frac{\partial P}{\partial t}=\Gamma (1+i\alpha )[-P-(1+i\alpha )DF]$$

3 $$\frac{\partial D}{\partial t}=b[\mu -D+\frac{1}{2}\left({F}^{* }P+F{P}^{* }\right)]$$

Here, the dynamical variables *F*, *P*, and *D* represent the scaled electric field and polarization envelopes, and the carrier density, respectively. The field and carrier dynamical rates, normalized with respect to the polarization dephasing time *τ*_*d*_, are respectively *σ* = *τ*_*d*_/*τ*_*p*_ (where *τ*_*p*_ is the photon lifetime) and *b* = *τ*_*d*_/*τ*_*e*_ (where *τ*_*e*_ is the carrier lifetime), while *Γ* corresponds to the scaled gain bandwidth. The parameter *α* denotes the linewidth enhancement factor. Finally, *η* and *t* are the dimensionless spatial and temporal variables, respectively. The scaling and the sign conventions follow the notation reported in^55^. Using this scaling, we convert the output power, time, and frequency into physical units, as presented in the figures of this work. The dynamical equations are complemented by the boundary condition of the ring cavity:

4 $$F(0,t)=\sqrt{R}F(L,t)$$

where *L* is the cavity length, and *R* is the power reflection coefficient. In this work we assume the limit value *R* = 1, i.e. a configuration similar to that reported in^25^.

Different from the Maxwell-Bloch equations for two-level systems, Eqs. (1)– (3) include essential aspects of the light-matter interaction characteristic of semiconductor lasers, such as the asymmetric frequency-dependent gain and refractive index and the coupling between field amplitude and phase, described by a non-zero *α* factor. These elements prove again a fundamental mechanism for the emergence of mode locking and, in this instance, of the HFCs.

With the final aim to describe the instabilities affecting a CW emission at threshold, we now study the conditions in which the medium coherently behaves in an oscillatory fashion. We thus assume that the field *F* is constant in space and monochromatic, i.e. the medium interacts with a CW at the reference frequency (the maximum of the laser unsaturated gain). This results in a linear system governed by equations for *P*, P^*^, and *D* whose short term perturbative dynamics is described by the complex eigenvalues *β* of the system coefficient matrix *A* (see Section III in the Supplemental Material). We highlight that an analogous procedure is adopted to estimate the Rabi frequency in a collection of two-level atoms^48,56^. Since it turns out that for *α* ≠ 0 two of these eigenvalues are complex conjugate and one is real, we identify the imaginary part of the complex solutions with a characteristic frequency associated with the oscillations of the material variables (*P* and *D*). We name Effective Rabi Frequency (ERF) this frequency in analogy with the Rabi Frequency of the two-level system.

**Fig. 1:**
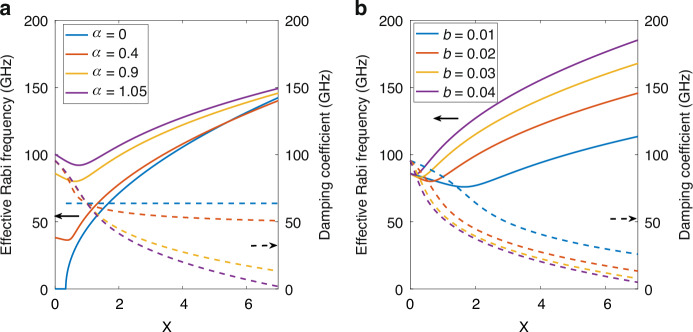
Effective Rabi frequency (solid) and damping coefficient (dashed) as a function of X = ∣F∣^2^ for different values of α and b. Figure (**a**) shows these quantities for different *α* values while Figure (**b**) shows them for different *b*. Solid and dashed curves of the same color indicate the same *α* (**a**) and *b* (**b**). The ERF and the damping coefficient are calculated as specified in the Supplemental Material. In both figures *Γ* = 0.06, corresponding to a gain bandwidth of ≃ 200 GHz. In (**a**) *b* = 0.02, while in (**b**) *α* = 0.9

In Fig. 1(a), we plot the ERF as a function of *X* = ∣*F*∣^2^ for different values of the *α* factor (solid lines). For *α* ≠ 0, the curve exhibits a decreasing trend at low field intensity before transitioning into a portion that grows as $\sqrt{X}$. As *α* diminishes, the decreasing portion progressively narrows until it vanishes completely at *α* = 0, where, as expected, the curve follows a pure $\sqrt{X}$ trend, characteristic of the Rabi Frequency for two-level system case^48^ (see also Section III in the Supplemental Material). The dashed curves in Fig. 1(a) show the *X*-dependence of the damping coefficient, given by the absolute value of the real part of *β*. For *X* > 1, the damping decreases with increasing *α*, nearly vanishing for large *X* and *α* = 1.05—a typical value for QCLs^17,42,57^. This supports the experimental observation of HFCs in these lasers, as will be clarified in the following. For *α* = 0, in the region where *ERF* = 0, the damping coefficient is not plotted, as no oscillations occur and damping is therefore not well-defined.

In Fig. 1(b), the ERF vs. *X* curve (solid) is plotted for varying *b*. The segment corresponding to a decrease of the ERF with the field intensity becomes more extended for larger *b*, i.e. for a faster carrier dynamics, and disappears in the two-level limit, where the system becomes an overdamped oscillator (*ERF* = 0).

**Fig. 2:**
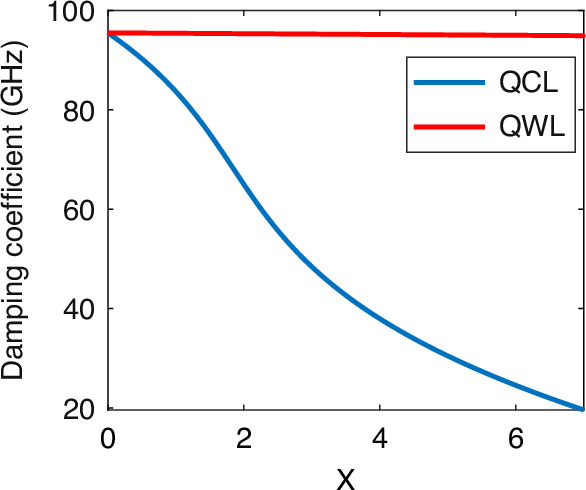
Damping coefficient as a function of *X* for a quantum cascade laser (QCL, blue) and a quantum well laser (QWL, red). The QCL parameters are *α* = 1.05 and *b* = 10^−2^, while the QWL parameters are *α* = 3 and *b* = 10^−4^. In both cases, *Γ* = 0.06

Moreover, faster carrier dynamics, associated with larger *b*, lead to reduced damping (dashed curves in Fig. 1(b)). This evidence is in agreement with the experimental observation of Rabi flopping in QCLs and QD amplifiers that are characterized by a similar effective carriers lifetime^58–60^. Consistently, in quantum well lasers (QWLs)—characterized by carrier lifetimes two to four orders of magnitude longer than those in QCLs— we verified that the damping is significantly higher. Figure 2 shows the damping coefficient as a function of *X* for the parameters of a QCL (blue curve) and a QWL (red curve). In the QCL case, the damping coefficient exhibits a pronounced decrease with increasing *X*. In contrast, for the QWL, it remains essentially constant and significantly higher than in the QCL, thereby inhibiting the observation of effective Rabi oscillations and HFCs in QWLs.

From these evidences we may thus expect that, in analogy to what happens for the Rabi frequency in the RNGH instability in two-level systems^48^, the ERF could have a role in CW instability and eventually in the emergence of OFCs. This was confirmed by performing the Linear Stability Analysis (LSA) of the CW solutions of Eqs. (1)–(3), whose expressions are provided in Section I of the Supplemental Material.

We observe that the CW corresponding to the minimum threshold, denoted as CW0 in the following, is that obtained by setting the wavenumber *k* = 0 in the steady state equations, i.e. the closest to the gain peak (which is the reference frequency)^24^.

To study the stability of the CW0 solution against the growth of undamped modes, we determine the eigenvalues *λ*_*n*_ of the Jacobian Matrix associated with the linear system for the perturbations that is obtained by seeking solutions to Eqs. (1)–(3) in the form $F=({F}_{0}+{\sum }_{n}\delta {F}_{n}{e}^{-i{k}_{n}\eta }{e}^{{\lambda }_{n}t}){e}^{-ik\eta +i\omega t}$, $P=({P}_{0}+{\sum }_{n}\delta {P}_{n}{e}^{-i{k}_{n}\eta }{e}^{{\lambda }_{n}t}){e}^{-ik\eta +i\omega t}$, and $D={D}_{0}+{\sum }_{n}\delta {D}_{n}{e}^{-i{k}_{n}\eta }{e}^{{\lambda }_{n}t}$, where ∣*δF*_*n*_∣ ≪ ∣*F*_0_∣, ∣*δP*_*n*_∣ ≪ ∣*P*_0_∣, *δD*_*n*_ ≪ *D*_0_ and *k*_*n*_ = 2*πn*/*L* are the Fourier modes. The details of the LSA are provided in Section II of the Supplemental Material. The parametric gain, defined as the maximum of the real part of *λ*_*n*_, can then be calculated. This is evaluated at a frequency *ν*_n_ = *ck*_*n*_ relative to the CW frequency and treated as a continuous variable (see the Supplemental Material).

**Fig. 3:**
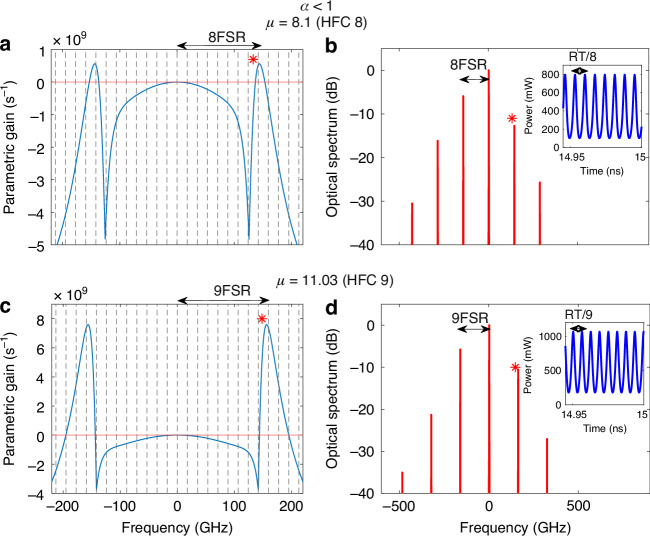
Harmonic combs of different orders, obtained for different values of the pump parameter *μ* and for *α* = 0.95. The other parameters are *Γ* = 0.06, L = 4.7 mm, *σ* = 1.6 × 10^−3^, and *b* = 0.014, with *τ*_*e*_ = 7 ps and *τ*_*d*_ = 0.1ps. The left panels (**a**, **c**) show the parametric gain, max(*Re*(*λ*_*n*_)), as a function of frequency (blue curve), the cavity modes (black dashed lines). The right panels (**b**, **d**) present the corresponding optical spectrum, obtained by numerically solving Eqs. (1)-(3), and associated with HFCs of order 8 (top row) and 9 (bottom). The effective Rabi frequency (red marker) is superimposed to both parametric gain and optical spectrum. The insets in panels (**b**, **d**) illustrate the numerically obtained temporal power trace of each HFC. RT = round trip time

Figure 3(a) (blue curve) shows an example of the parametric gain numerically computed for typical parameters of a THz QCL and pump parameter *μ* = 8.01 (see figure caption). The frequency corresponding to the peak of the parametric gain coincides with the laser cavity mode (black dashed lines) spaced by 8 FSRs from that of the CW0 (*k*_0_ = 0). Remarkably, the ERF (red marker) closely matches the parametric gain peak frequency. Solving numerically Eqs.(1)–(3) for the same parameters as in Fig. 3(a), shows that for the same parameters the CW is unstable and the system actually operates in a 8th-order harmonic comb regime (Fig. 3(b)). Hence this suggests a connection between HFCs formation and the resonance between the ERF and one of the cavity modes. We emphasize that this result is in agreement with experimental observations of self-starting HFCs in ring QCLs operating in the THz region^35,61^. In particular, the transition from a CW regime to a harmonic comb state with increasing QCL pump current has been reported in this class of devices^35^.

Increasing *μ* to 11.03 shifts the parametric gain peak frequency, which aligns with the cavity mode spaced 9 FSRs from the central frequency (Fig. 3(c)). The ERF (red marker) also shifts, following the parametric gain peak. Consistent with the previous case, numerical simulations confirm the predictions of the LSA and the ERF, resulting in the numerical observation of a 9th-order HFC, as depicted in Fig. 3(d). The scenario emerging from our theoretical analysis is also consistent with experiments in FP QCLs, where an increase in the order of self-starting harmonic combs with increasing bias current is reported^31,32^.

**Fig. 4:**
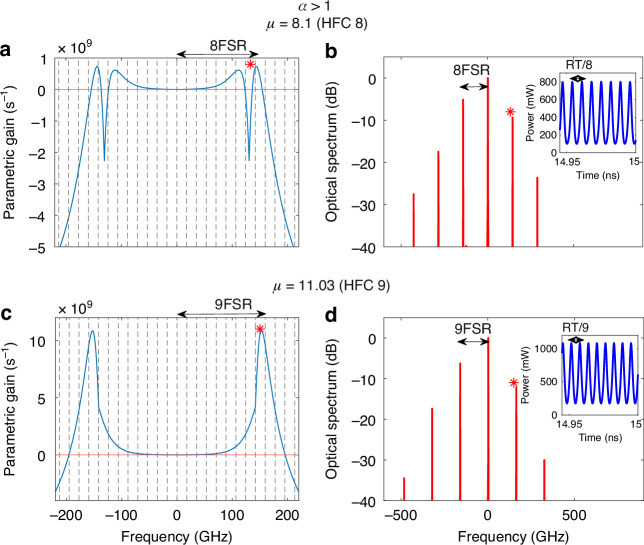
Harmonic combs of different orders for *α* = 1.01. The left panels (**a**, **c**) show the parametric gain as a function of frequency (blue curve) and the cavity modes (black dashed lines). The right panels (**b**, **d**) present the optical spectrum. The insets in panels (**b**, **d**) illustrate the temporal power trace of each HFC. The effective Rabi frequency (red marker) is superimposed to both parametric gain and optical spectrum. The parameters (except for *α*) and the quantities plotted are the same as in Fig. 3

The results presented in Fig. 3 were obtained for *α* < 1. A similar behavior is also observed for *α* > 1, although the shape of the parametric gain as a function of the frequency (and consequently the type of instability) differs from that in the *α* < 1 case (see Fig. 4). For *α* < 1, which is close to the two-level system case (formally corresponding to *α* = 0), the CW instability is analogous to the RNGH characterized by a balloon of unstable vectors *k*_n_^48,54^ with a lower limit different from zero and with a maximum close to the ERF; instead for *α* > 1, which represents a more realistic case for a QCL, especially in the mid-IR range^14,57^, the CW instability is closer to the Benjamin-Feir or phase instability since it corresponds to a balloon of unstable wave vectors *k*_*n*_ with a lower limit equal to *k*_0_ = 0 and with a maximum close to the ERF. The BF instability is typical of the CGLE^53^ that describes well a unidirectional ring QCL close to threshold^25,28^.

In this instance (with *α* > 1), we could observe that at lower values of *μ* compared to those in Fig. 4, this Benjamin-Feir-like instability leads to the formation of OFCs associated with the presence of homoclons in the cavity, as predicted and experimentally reported in^25^.

In some other cases we observe, for the same pump *μ*, two distinct HFCs with different spacing and corresponding to the destabilization of different CWs, depending on how the pump parameter is scanned. Moreover, transitions between these solutions are also observed within a single simulation through a transient (see Fig. S2 in Supplemental Material). A similar behavior has also been experimentally reported^36^, though its interpretation remained unclear. In this work, we have chosen to present cases for values of *μ* where a single harmonic comb state is stably observed.

**Fig. 5:**
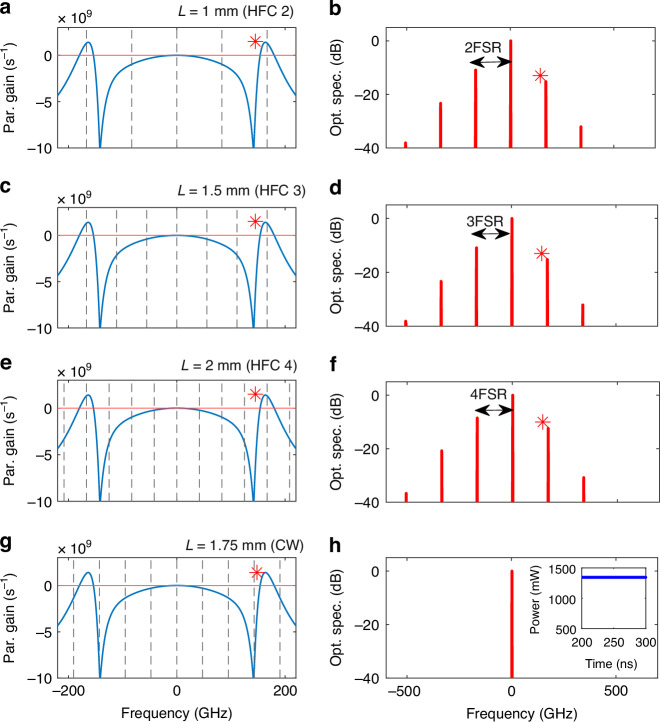
Different regimes obtained by scanning the cavity length *L* at fixed *α* = 0.92. For *L* = 1, 1.5, 2 mm, HFCs of different order are reported in simulations, while for *L* = 1.75 mm, a CW is obtained. The other parameters are *Γ* = 0.06, *μ* = 7.9, *σ* = 1.6 × 10^−3^, and *b* = 0.02, with *τ*_*e*_ = 5 ps and *τ*_*d*_ = 0.1ps. The left panels (**a**, **c**, **e**, **g**) show the parametric gain as a function of the frequency. The right panels (**b**, **d**, **f**, **h**) present the simulated optical spectrum. The inset in panel (**h**) illustrates the simulated temporal power trace for the CW regime. The power traces for the HFCs are very similar to those in previous figures, therefore they are omitted. The red markers indicate the estimated ERF

To further validate our interpretation, we then investigate how the properties of the formation of HFCs as the cavity length varies, focusing first on the case of *α* < 1 (Fig. 5). For *L* = 1 mm and the other parameters set as reported in the figure caption, the parametric gain peak and the ERF are both located near the resonator mode at 2 FSRs from the CW frequency, consistent with numerical simulations showing a second-order HFC regime (Figs.5(a)-(b)). Increasing the cavity length to *L* = 1.5 mm reduces the free spectral range, shifting the parametric gain peak and ERF to align with a mode 3 FSRs from the central frequency. Simulations confirm the emergence of a third-order harmonic comb (Figs.5(c, d)). Similarly, for *L* = 2 mm, the gain peak and ERF coincide with a mode 4 FSRs from the central frequency, corresponding to a fourth-order harmonic comb (Figs. 5(e, f)). Remarkably, for *L* = 1.75 mm—intermediate between 1.5 mm and 2 mm—the peak of the parametric gain lies between two adjacent cavity modes (Fig. 5(g)), and no mode has a positive parametric gain. Consistently, numerical simulations reveal a stable CW regime instead of a harmonic comb (Fig. 5(h)). In this case as well, the ERF remains close to the peak of the parametric gain. This counterexample further corroborates the interpretation that HFCs formation arises from a resonance mechanism. Furthermore, we observe similar behavior by changing *L* for *α* > 1 albeit with the differences in the parametric gain lineshape mentioned above (see Section IV in the Supplemental Material).

Therefore, further implications of a non-zero LEF emerge, establishing a critical distinction between semiconductor and two-level system. Notably, the value of *α* dictates the shape of the parametric gain lines and the nature of the instability that underpins the formation of frequency combs.

## Discussion

The presented results highlight the significant predictive power of the LSA in forecasting HFCs formation in a semiconductor laser. Furthermore, they allow to interpret the self-starting formation of HFC states as a resonance phenomenon that occurs when an intrinsic frequency of the system (the ERF) approaches one of the laser cavity modes. While this is reminiscent of the role played by the Rabi frequency in the onset of the RNGH instability in lasers with an active medium consisting of a collection of two-level systems^48^, the theory we develop here applies to any semiconductor laser exhibiting multimode emission or frequency comb formation, with a significantly non-zero LEF. Moreover, our study shows that in ultrafast lasers such as QCLs, the oscillations occurring at ERF are characterized by lower damping than in conventional quantum-well lasers and can therefore trigger the instability of CW emission that leads to the formation of multimode regimes and frequency combs.

Our results also clarify that the reduced model based on the Complex Ginzburg–Landau equation —widely used to study ring QCLs^25,29,55^—does not provide a complete description of the multimode instability. While it predicts only a long-wavelength (BF type) instability, the ESMBEs model reveals that this is just one of the possible scenarios, occurring specifically when *α* > 1. When *α* < 1, by contrast, the system undergoes an instability of the RNGH type.

Furthermore, our theoretical results are in agreement with the experimental observation of HFCs in THz ring QCLs^35,61^. The increase of the comb harmonic order with increasing pump parameter reported in this study is also consistent with experiments in FP QCLs^31,32^.

In conclusion, we have provided a physical interpretation for the formation of self-starting HFCs in QCLs, recognized as one of the main outstanding open questions in the field of QCLs frequency combs^17,42^. Understanding the physical mechanism behind the spontaneous formation of HFCs paves the way for a paradigm shift in the procedure for their generation and control. Whereas previous efforts have primarily focused on inducing and engineering these states using external interventions such as optical and electrical injection or a suitable reflectivity discontinuity or defects^33,37,40^, this work suggests alternative ways to tailor their properties. These include acting on bias conditions or on the QCL cavity design to tune the resonator mode spacing, for example, using mechanical^62^ or electro-optic^63^ methods. Moreover, the analytical approaches developed in this work—namely the estimation of the ERF and the linear stability analysis—can serve as complementary tools for the design of QCL structures specifically tailored for the generation of HFC states. Due to the suitability of QCLs for on-chip integration, our findings could accelerate the development of HFC-based photonic applications. These include mid-IR and THz pulse generation, microwave and THz photonics for radio-frequency waveform synthesis, and the generation of THz carriers for next-generation wireless networks^36,64,65^—fields that have so far remained in their infancy due to the complexity of experimental platforms required for harmonic combs.

## Methods

### Effective semiconductor Maxwell-Bloch equations

The Effective Semiconductor Maxwell-Bloch Equations (ESMBEs) used in this work describe the dynamics of a multimode Quantum Cascade Laser (QCL) in a unidirectional ring configuration^24^. A normalized form of the ESMBEs is adopted, following the normalization reported in^55^. This model captures several key properties of semiconductor materials, including asymmetric gain and refractive index profiles, a nonzero linewidth enhancement factor, and the dependence of the optical susceptibility on the carrier density. The dynamical variables in the set of ESMBEs are the electric field, polarization, and carrier density. The ESMBEs are integrated through a time-domain traveling-wave method, which employs an advanced finite-difference scheme in both temporal and spatial domains^13^.

### Linear stability analysis

A linear stability analysis of the continuous-wave solutions of the ESMBEs is carried out in this work. The procedure follows the approach in^48^, where time-dependent perturbations of the dynamical variables are introduced, leading to a linear system whose eigenvalues are obtained by solving the secular equation. The full derivation is provided in Section II of the Supplemental Material.

## Supplementary information

Effective Rabi frequency in ultrafast semiconductor lasers: self-starting harmonic frequency combs - Supplementary Materials

## Data Availability

The authors state that data generated in this study are provided within the article and Supplemental Material. All data are available from the corresponding author upon reasonable request.

## Code availability

The code used in this study is available from the corresponding author upon reasonable request.
