# Supplementary material for: Effective Rabi frequency in ultrafast semiconductor lasers: self-starting harmonic frequency combs
Source: Light Sci Appl. 2026 Jul 10;15:312. doi: 10.1038/s41377-026-02342-1 (PMC13354799; doi:10.1038/s41377-026-02342-1)
Supplement: Supplementary file 1 — Effective Rabi frequency in ultrafast semiconductor lasers: self-starting harmonic frequency combs - Supplementary Materials [file 41377_2026_2342_MOESM1_ESM.pdf]

Effective Rabi frequency in ultrafast semiconductor lasers: self-starting harmonic frequency combs – Supplementary materials

Carlo Silvestri,[1] Franco Prati,[2] Massimo Brambilla,[3] Mariangela Gioannini,[4] and Lorenzo Luigi Columbo[4]

[1]*Institute of Photonics and Optical Science (IPOS), School of Physics, The University of Sydney, NSW 2006, Australia*

[2]*Dipartimento di Scienza e Alta Tecnologia, Università dell'Insubria, 22100 Como, Italy*

[3]*Dipartimento Interateneo di Fisica, Politecnico di Bari and CNR-IFN, UOS Bari, Italy*

[4]*Dipartimento di Elettronica e Telecomunicazioni, Politecnico di Torino, 10129 Torino, Italy*

I. CONTINUOUS WAVE SOLUTIONS OF THE EFFECTIVE SEMICONDUCTOR MAXWELL-BLOCH EQUATIONS

Let us consider the ESMBEs for the ring configuration:

$$\frac{\partial F}{\partial \eta}+\frac{\partial F}{\partial t} = -\sigma\left(F+P\right) \tag{S1}$$

$$\frac{\partial P}{\partial t} = -\Gamma(1+i\alpha)^2 FD-\Gamma(1+i\alpha)P \tag{S2}$$

$$\frac{\partial D}{\partial t} = b\left[\mu-D+\frac{1}{2}\left(F^*P+FP^*\right)\right]. \tag{S3}$$

Following the approach presented in [1], we seek solutions of the form $F=F_0e^{-ik\eta+i\omega t}$, $P=P_0e^{-ik\eta+i\omega t}$, $D=D_0$. We then obtain:

$$-ikF_0+i\omega F_0 = -\sigma(F_0+P_0) \tag{S4}$$

$$i\omega P_0 = -\Gamma(1+i\alpha)^2F_0D_0-\Gamma(1+i\alpha)P_0 \tag{S5}$$

$$0 = \mu-D_0+\frac{1}{2}\left(F_0^*P_0+F_0P_0^*\right). \tag{S6}$$

From Eq. (S5) we can write an expression for P_0:

$$P_0=-\frac{\Gamma(1-\alpha^2)+2i\alpha\Gamma}{\Gamma+i(\omega+\alpha\Gamma)}D_0F_0=\left[-H_1(\omega)+iH_2(\omega)\right]D_0F_0 \tag{S7}$$

with

$$H_1(\omega) = \frac{\Gamma^2(1-\alpha^2)+2\alpha\Gamma(\omega+\alpha\Gamma)}{\Gamma^2+(\omega+\alpha\Gamma)^2} \tag{S8}$$

$$H_2(\omega) = \frac{-2\alpha\Gamma^2+\Gamma(1-\alpha^2)(\omega+\alpha\Gamma)}{\Gamma^2+(\omega+\alpha\Gamma)^2}. \tag{S9}$$

Substituting Eq. (S7) in Eqs. (S4) and (S6) we obtain:

$$F_0\left(ik-i\omega-\sigma\right) = \sigma\left[-H_1(\omega)+iH_2(\omega)\right]D_0F_0 \tag{S10}$$

$$0 = \mu-D_0-H_1(\omega)|F_0|^2D_0. \tag{S11}$$

Dividing Eq. (S10) by F_0 and splitting it into real and imaginary parts, we obtain from the real part:

$$D_0=\frac{1}{H_1(\omega)} \tag{S12}$$

and from the imaginary part:

$$\omega=k-\sigma\frac{H_2(\omega)}{H_1(\omega)}. \tag{S13}$$

Eq. (S11) gives an expression for D_0

$$D_0 = \frac{\mu}{1 + |F_0|^2 H_1(\omega)} \tag{S14}$$

which, combined with Eq. (S12), allows to write the intensity as

$$|F_0|^2 = \mu - \frac{1}{H_1(\omega)}\,. \tag{S15}$$

We highlight that Eq. (S15) describes the dependence of the field intensity on the pump parameter, while Eq. (S13) represents the dispersion relation, which, as can be noticed, is a nonlinear equation.

II. LINEAR STABILITY ANALYSIS

In this section we perform the linear stability analysis of the CW solutions Eqs. (S7), (S12), (S15), introducing time dependent perturbations for the dynamical variables F, P, and D [2, 3]:

$$F = (F_0 + \delta F)e^{-ik\eta + i\omega t} \tag{S16}$$
$$P = (P_0 + \delta P)e^{-ik\eta + i\omega t} \tag{S17}$$
$$D = D_0 + \delta D. \tag{S18}$$

We substitute Eqs. (S16)–(S18) into Eqs. (S1)–(S3), drop the nonlinear terms and expand the generic dynamical variable perturbation δX as:

$$\delta X = \sum_n \delta X_n e^{-ik_n\eta} e^{\lambda_n t} \tag{S19}$$

where k_n represent the wavenumbers of the mode expansion for each of the dynamical variables. Then, for each mode index n, we obtain a closed subset of equations

$$\delta F_n[\sigma - i(k - \omega + k_n) + \lambda_n] + \sigma\delta P_n \;=\; 0 \tag{S20}$$
$$\delta F^*_{-n}[\sigma + i(k - \omega - k_n) + \lambda_n] + \sigma\delta P^*_{-n} \;=\; 0 \tag{S21}$$
$$\delta P_n[i\omega + \Gamma(1 + i\alpha) + \lambda_n] + \Gamma(1 + i\alpha)^2(D_0\delta F_n + F_0\delta D_n) \;=\; 0 \tag{S22}$$
$$\delta P^*_{-n}[-i\omega + \Gamma(1 - i\alpha) + \lambda_n] + \Gamma(1 - i\alpha)^2(D_0\delta F^*_{-n} + F^*_0\delta D_n) \;=\; 0 \tag{S23}$$
$$\delta D_n(\lambda_n + b) - \frac{b}{2}\left(P_0\delta F^*_{-n} + F^*_0\delta P_n + F_0\delta P^*_{-n} + P^*_0\delta F_n\right) \;=\; 0\,. \tag{S24}$$

From Eqs. (S20)–(S24), we can construct the matrix M_{λ_n} of the linearized system:

$$M_{\lambda_n} = \begin{pmatrix} \sigma - i(k - \omega + k_n) + \lambda_n & 0 & \sigma & 0 & 0 \\ 0 & \sigma + i(k - \omega - k_n) + \lambda_n & 0 & \sigma & 0 \\ \Gamma(1 + i\alpha)^2 D_0 & 0 & i\omega + \Gamma(1 + i\alpha) + \lambda_n & 0 & \Gamma(1 + i\alpha)^2 F_0 \\ 0 & \Gamma(1 - i\alpha)^2 D_0 & 0 & -i\omega + \Gamma(1 - i\alpha) + \lambda_n & \Gamma(1 - i\alpha)^2 F^*_0 \\ -\frac{b}{2}P^*_0 & -\frac{b}{2}P_0 & -\frac{b}{2}F^*_0 & -\frac{b}{2}F_0 & b + \lambda_n \end{pmatrix}. \tag{S25}$$

The eigenvalues λ_n are determined by solving the characteristic equation $\det(M_{\lambda_n}) = 0$.

III. EFFECTIVE RABI FREQUENCY

We assume that the field F is constant and monochromatic with a frequency coincident with the maximum of the unsaturated gain and consider the dynamical equations for P, P^*, and D

$$\frac{dP}{dt} = -\Gamma(1 + i\alpha)^2 DF - \Gamma(1 + i\alpha)P \tag{S26}$$
$$\frac{dP^*}{dt} = -\Gamma(1 - i\alpha)^2 DF - \Gamma(1 - i\alpha)P^* \tag{S27}$$
$$\frac{dD}{dt} = b\mu - bD + \frac{b}{2}\left(F^*P + FP^*\right). \tag{S28}$$

The dynamics of this linear system is described by the complex eigenvalues β solutions of the characteristic equation $\det(M_\beta) = 0$ with

$$M_\beta = \begin{pmatrix} \beta + \Gamma(1+i\alpha) & 0 & \Gamma(1+i\alpha)^2 F \\ 0 & \beta + \Gamma(1-i\alpha) & \Gamma(1-i\alpha)^2 F^* \\ -\frac{b}{2}F^* & -\frac{b}{2}F & \beta + b \end{pmatrix} . \tag{S29}$$

The cubic characteristic equation is

$$\beta^3 + (2\Gamma + b)\beta^2 + \Gamma\left[\Gamma(1+\alpha^2) + b(2 + X - X\alpha^2)\right]\beta + b\Gamma^2(1+\alpha^2)(1+X) = 0 \tag{S30}$$

where we set $X = |F|^2$. As mentioned in the main text, Eq. (S30) has in general two complex-conjugate solutions and one real solution. Therefore, we identify the Effective Rabi frequency (ERF) with the absolute value of the imaginary part of the complex solutions. In physical units, the expression of the Rabi frequency is ERF= $|Im(\beta)|/(2\pi\tau_d)$. The damping coefficient is defined for the complex solutions as $|\mathrm{Re}(\beta)|/(2\pi\tau_d)$.
If $\alpha = 0$ (two-level atoms) the characteristic equation can be factorized as

$$(\beta + \Gamma)\left[\beta^2 + (\Gamma + b)\beta + b\Gamma(1+X)\right] = 0 \tag{S31}$$

whose solutions are

$$\beta_1 = -\Gamma\,, \qquad \beta_\pm = \frac{1}{2}\left[-\Gamma - b \pm \sqrt{(\Gamma - b)^2 - 4b\Gamma X}\right] . \tag{S32}$$

The latter are complex if $X > (\Gamma - b)^2/(4b\Gamma)$ and the associated ERF is

$$\mathrm{Im}(\beta_+) = \sqrt{4b\Gamma X - (\Gamma - b)^2} \tag{S33}$$

as for two-level lasers at resonance [4]. At the laser threshold ($X = 0$) the characteristic equation (S30) can be factorized as well

$$(\beta + b)\left[\Gamma^2\alpha^2 + (\Gamma + \beta)^2\right] = 0\,. \tag{S34}$$

The solutions are

$$\beta_1 = -b\,, \qquad \beta_\pm = -\Gamma \pm i\Gamma\alpha\,. \tag{S35}$$

and the associated ERF is

$$\mathrm{Im}(\beta_+) = \Gamma\alpha\,. \tag{S36}$$

This explains the behaviour reported in Fig. 1(a) of the main text, where the ERF at $X = 0$ increases proportionally to α and the curves representing the damping coefficient start all from the same value since Γ is kept constant.

IV. SCAN OF THE CAVITY LENGTH FOR $\alpha > 1$

In Fig. S1 we report the HFCs obtained for $\alpha > 1$ as the cavity length varies. This completes the discussion done in the main text and the results reported in Fig. 5.

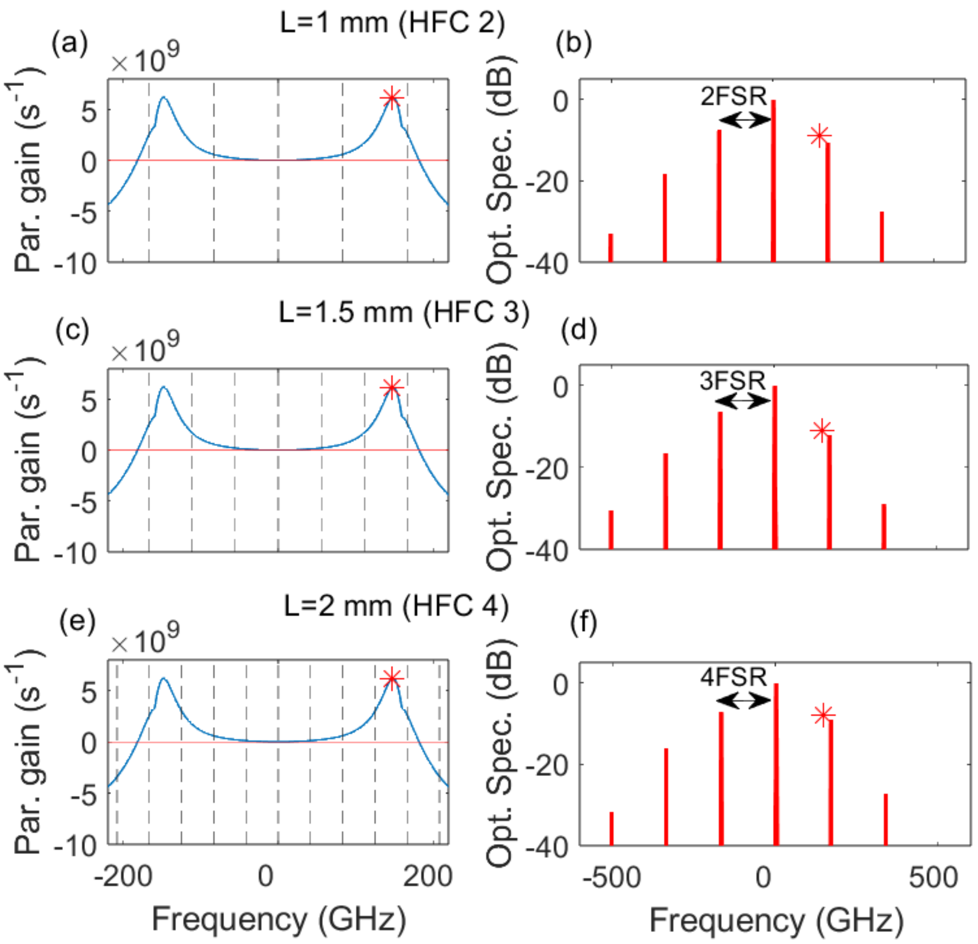

FIG. S1. Harmonic frequency combs of different order obtained by scanning the cavity length L at fixed $\alpha = 1.05$. The other parameters are $\Gamma = 0.06$, $\mu = 7.9$, $\sigma = 1.6 \times 10^{-3}$, and $b = 0.02$. Parametric gain as a function of the frequency (panels (a), (c), (e)), and simulated optical spectra (panels (b), (d), (f)). The red markers represent the estimated ERF. Each figure in the same column has the same scale on the horizontal axis.

V. TRANSITION BETWEEN TWO DIFFERENT HARMONIC STATES

We present here a simulation example in which a transition between two different harmonic states is observed for the same value of the pump parameter (see Fig. S2). At the beginning of the simulation, the laser starts emitting in a continuous wave (CW) at $f_c = 2.5$ GHz from the reference frequency, which is the fundamental CW corresponding to $k = 0$. This CW then destabilizes in favor of the formation of a 7th-order harmonic frequency comb (HFC) (see insets in the top left). We verified that the peak of the parametric gain and the ERF are in proximity to the cavity mode located 7 FSRs away from the reference frequency, as shown in Fig. S3. After a few nanoseconds the simulation shows (Fig. S2), a transition to an irregular transient, lasting several tens of nanoseconds. Then, the system settles into a new 11th-order harmonic comb regime, with a central frequency of $f_c = -64$ GHz. Therefore, this second HFC corresponds to the destabilization of a different CW mode than the one associated with the harmonic comb observed in the first part of the simulation.

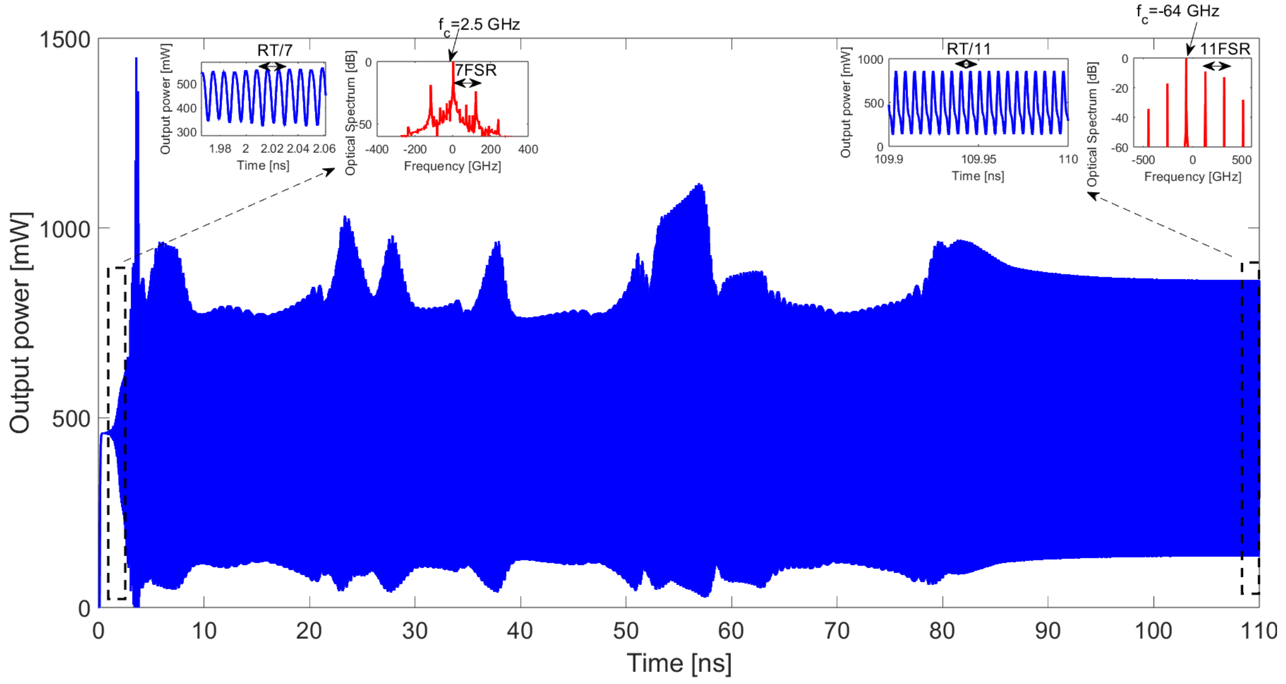

FIG. S2. Numerical simulation initially showing the formation of a transient 7th order HFC (insets on top left), followed by an irregular transient and the formation of a regular steady state 11th order HFC (insets on top right). Parameters: $\alpha = 1.05$, $\Gamma = 0.06$, $b = 0.014$, and $\mu = 5.9$, $\sigma = 1.6 \times 10^{-3}$.

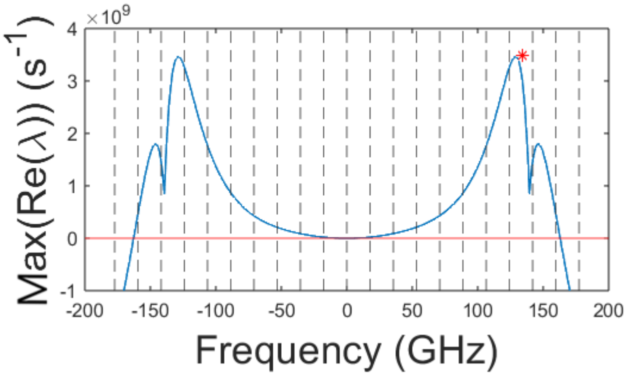

FIG. S3. Parametric gain and ERF (red marker) for the fundamental CW ($k = 0$) with the parameters of Fig. S2.

REFERENCES

[1] L. L. Columbo, S. Barbieri, C. Sirtori, and M. Brambilla, Dynamics of a broad-band quantum cascade laser: from chaos to coherent dynamics and mode-locking, Opt. Express **26**, 2829 (2018).

[2] L. Lugiato, F. Prati, and M. Brambilla, *Nonlinear Optical Systems* (Cambridge University Press, 2015).

[3] L. L. Columbo, P. Bardella, and M. Gioannini, Self-pulsing in single section ring lasers based on quantum dot materials: theory and simulations, Opt. Express **26**, 19044 (2018).

[4] H. C. Torrey, Transient nutations in nuclear magnetic resonance, Phys. Rev. **76**, 1059 (1949).
